# Directly observed therapy for HCV with glecaprevir/pibrentasvir alongside opioid substitution in people who inject drugs—First real world data from Austria

**DOI:** 10.1371/journal.pone.0229239

**Published:** 2020-03-10

**Authors:** Caroline Schmidbauer, Raphael Schubert, Angelika Schütz, Cornelia Schwanke, Julian Luhn, Enisa Gutic, Roxana Pirker, Tobias Lang, Thomas Reiberger, Hans Haltmayer, Michael Gschwantler

**Affiliations:** 1 Department of Internal Medicine IV, Wilhelminenspital, Vienna, Austria; 2 Division of Gastroenterology and Hepatology, Department of Internal Medicine III, Medical University of Vienna, Vienna, Austria; 3 Vienna HIV & Liver Study Group, Vienna, Austria; 4 Ambulatorium Suchthilfe Wien, Suchthilfe Wien gGmbH, Vienna, Austria; 5 Sigmund Freud University, Vienna, Austria; Medizinische Fakultat der RWTH Aachen, GERMANY

## Abstract

**Background:**

Directly acting antivirals (DAA) against hepatitis C virus (HCV) infection have facilitated sustained virologic response (SVR) rates >90% in clinical studies. Yet, real life data regarding DAA treatment in people who inject drugs (PWIDs) are scarce. We evaluated the effectiveness of glecaprevir/pibrentasvir (G/P) in difficult-to-treat PWIDs with presumed high risk of non-adherence to DAA therapy using the concept of directly observed therapy involving their opioid substitution therapy (OST) facility.

**Methods:**

N = 145 patients (m/f: 91/54; median age: 41.1 (IQR 19.5) years; HCV-genotype (GT) 1/2/3/4: 82/1/56/5, GT3: 38.6%; cirrhosis: n = 6; 4.1%) treated with G/P were included. PWIDs at high risk for non-adherence to DAA therapy received HCV treatment together with their OST under the supervision of medical staff ("directly observed therapy", DOT). The effectiveness of G/P given as DOT in PWIDs with presumed high risk of non-adherence to DAA therapy was compared to patients with suspected “excellent compliance” in the "standard setting" (SS) of G/P prescription at a tertiary care center and self-managed G/P intake at home. Treatment duration was 8–16 weeks according to the G/P drug label.

**Results:**

DOT-patients (n = 74/145; 51.0%) were younger than SS-patients (median 38.7, IQR 12.5 vs. median 50.6, IQR 20.3 years), all had psychiatric co-morbidities and most had a poor socioeconomic status. 50/74 (67.6%) reported ongoing intravenous drug use (IDU). SVR was achieved in n = 70/74 (94.6%) patients with n = 3 being lost to follow-up (FU) and n = 1 showing nonresponse to therapy.

SS-patients achieved SVR in 97.2% (69/71) with n = 1 patient being lost to FU and n = 1 patient with GT3 showing HCV relapse.

**Conclusion:**

G/P given as DOT along with OST in PWIDs with high risk of non-adherence to DAA therapy resulted in similarly high SVR rates (94.6%) as in patients with presumed “excellent compliance” under standard drug intake.

## Lay summary

Direct acting antivirals cure hepatitis C in >90% of all patients. People who inject drugs (PWIDs) represent a relevant at-risk population for hepatitis C infection and transmission, yet treatment efficacy is often limited by poor adherence to clinical visits and drug intake. Our real world results showed that PWIDs with high risk of non-adherence to DAA therapy receiving their hepatitis C medication together with opioid substitution under the direct supervision of medical staff achieve excellent HCV cure rates.

## Introduction

Hepatitis C virus (HCV) infection is a leading cause for cirrhosis, liver-related complications, hepatocellular carcinoma (HCC), liver transplantation and death worldwide. HCV treatment at an early stage of the disease can prevent the progression to cirrhosis and the development of liver-related complications, especially in patients infected at a young age[1,2].

Modern directly acting antiviral (DAA) therapy for HCV facilitates virological cure at a favorable safety and drug tolerability profile in nearly all patients[3–8]. The latest pangenotypic treatment regimens induce excellent SVR (sustained virologic response) rates independent of HCV-genotype (GT), even in former difficult-to-treat populations including patients with cirrhosis, pretreated subjects and HIV-coinfected patients[9]. The consecutive decrease in HCV-associated HCC and decompensated cirrhosis lead to a recomposition of liver transplant waitlists with an increased donor organ availability for patients suffering from other entities of liver disease[10]. Unfortunately, in many countries of the world access to pangenotypic DAA therapy is still limited by the high treatment costs[11].

The 2015 Agenda for Sustainable Development committed to combating viral hepatitis and the WHO provided models for the elimination of HCV as a public health threat[12,13]. The suggested strategies in order to reduce HCV transmission include the overall achievement of blood and injection safety coverages of 100% and 90%, respectively, as well as a specific increase in syringe/needle exchange set supplies provided for PWIDs from 20 (2015) to 300 (2030) sets per patient per year. Furthermore, an increase in HCV diagnosis from <5% (2015) to 90% (2030) and in treatment uptake from <1% (2015) to 80% of all eligible patients (2030) are necessary to achieve the goal of HCV elimination. Additionally, HBV (hepatitis B virus) and HCV incidence shall be reduced by 90% from 6–10 million (2015) until 2030[12,14–16].

The recent reduction or removal of reimbursement-restrictions concerning DAA therapy in Europe due to governmental programs and financial competition between pharmaceutical companies opened up new opportunities concerning the realization of these elimination targets[17,18]. One pangenotypic DAA combination that has recently become available in Europe is glecaprevir/pibrentasvir (G/P). Numerous phase III-studies have shown its excellent effectiveness and tolerability across various patient populations including HIV-coinfected patients, individuals with renal impairment or other relevant comorbidities as well as patients with a history of previous treatment failure[19–27].

Yet, real world data on G/P are still scarce, especially concerning PWIDs with and without ongoing injection drug use (IDU)[19,21,28]. Furthermore, there are nearly no data concerning difficult-to-treat PWIDs who are reluctant to attend tertiary care centers and have a high risk of non-adherence to therapy. For these patients we used a specifically tailored approach to treat chronic hepatitis C and compared effectiveness of G/P in this difficult-to-treat PWID population with the outcome of patients treated in the standard setting of a hepatological center. While recent analyses of the German Hepatitis C-Registry showed promising results concerning the safety and effectiveness of G/P[28], this is, to our knowledge, the first analysis of real world data on G/P from Austria. The results may serve as a basis for further HCV-microelimination strategies.

## Patients and methods

All DAA-naïve patients with chronic hepatitis C, 18 years of age or older, who started treatment with G/P at our center (comprising a tertiary care hospital and a low-threshold facility in Vienna) between October 2017 and December 2018 were included in this analysis.

### Setting of treatment

In Vienna there is a large population of about 6.500 PWIDs included in a nationwide OST program, receiving their OST on a daily or weekly basis at a pharmacy or a low-threshold facility. It is estimated that nearly one third of these patients suffer from chronic hepatitis C. According to our observations, a considerable percentage of PWIDs are reluctant to attend the outpatient clinic at a tertiary care hospital and hence cannot be treated there. We therefore built up a cooperation between the tertiary care center Wilhelminen Hospital Vienna and the Ambulatorium Suchthilfe Wien–a low-threshold drug treatment facility in Vienna. A second outpatient hepatitis clinic was established at the Ambulatorium Suchthilfe Wien, where patients were seen by an experienced hepatologist (M.G.) and all equipment for pretreatment evaluation of patients including laboratory tests and Fibroscan^®^ was available. This low-threshold facility combines HCV screening and treatment for PWIDs by offering an integrative management in a multidisciplinary setting that includes hepatological and psychiatric services as well as OST distribution and support by social workers.

In a high percentage of PWIDs on OST, especially in those with ongoing intravenous drug use, the team of physicians considered it to be unlikely for the patients to regularly ingest their HCV medication if a package for one month was handed to them for self-administration at home. To optimize adherence, this subgroup of PWIDs was treated according to our concept of “directly observed therapy” (DOT): Patients received their antiviral therapy together with OST under direct observation of a pharmacist, physician or nurse at a pharmacy or at the Ambulatorium Suchthilfe Wien on a once-daily basis (i.e. from Monday to Saturday and only the doses for Sunday were handed to the patient for self-administration at home) or, in a few cases, on a once-weekly, twice-weekly or thrice-weekly basis (“directly observed therapy group”).

Patients without a history of IDU and PWIDs in whom compliance was considered to be excellent were treated in the "standard setting" (SS) of the outpatient clinic of Wilhelminen Hospital Vienna: They received prescriptions for antiviral therapy every month and were only seen for routine laboratory tests at the outpatient clinic (“standard setting therapy group”).

The assignment to the different treatment groups (SS vs. DOT) was made by an interdisciplinary team consisting of an experienced hepatologist (M.G.), three addiction medicine specialists and the head nurse of the institution. The decision was based on the assumed likelihood of each individual patient to remain adherent to DAA-therapy if a monthly supply was handed to them for self-administration at home, as perceived by each member of the team. Every patient was seen by the entire team and the assignment to either of the treatment groups was made following a discussion among all team members. The frequency of visits to the drug distributing facility (daily vs. once, twice or three times a week) was based on the preceding frequency of OST dispensation: In order to maintain the previously well-established setting of directly observed OST-distribution, no changes were made in the preexisting drug distribution settings. The rationale behind this approach was that patients would not forget the ingestion of their OST, which is why simultaneous DAA and OST administration might ensure DAA adherence and hence HCV cure.

### Pretreatment workup of patients

Pretreatment evaluation of patients was done at the outpatient clinic of Wilhelminen Hospital Vienna or Ambulatorium Suchthilfe Wien and included detailed history, physical examination, abdominal sonography, standard laboratory testing, measurement of the serum HCV-RNA level, assessment of HCV GT and stage of liver fibrosis. In PWIDs on OST treated according to the concept of directly observed therapy the socioeconomic status was characterized by recording social relationships, housing status, employment status and criminal record.

Serum HCV-RNA was measured using the COBAS TaqMan HCV Quantitative Test, version 2.0 (Roche), with a lower limit of quantification of 15 IU/mL. The HCV GT and subtype were determined by use of the VERSANT HCV Genotype 2.0 Assay (Siemens). Prior to treatment, fibrosis stage was assessed by transient elastography using the Fibroscan^®^ 502 Touch device with the M-probe (Echosens, Paris, France) and classified according to the METAVIR scoring system (F0 = no fibrosis; F1 = portal fibrosis without septa; F2 = portal fibrosis and few septa; F3 = numerous septa without cirrhosis; F4 = cirrhosis). Cut-off values for liver stiffness (LS) were defined as 7.1 kPa for F ≥ 2, 9.5 kPa for F ≥ 3 and 12.5 kPa for F = 4[29–32]. Only procedures with 10 successful measurements of liver stiffness with an IQR < 30% were considered reliable.

In patients whose LS could not successfully be assessed by transient elastography, evaluation of fibrosis stage was performed by calculation of the APRI score (applied ULN for AST: 35 IU/L (female) and 50 IU/L (male); F0/F1: APRI ≤ 0.5, F4: APRI > 1.5)[29,31–33].

### Definition of ongoing intravenous drug use and measures to prevent reinfection

Ongoing IDU was defined as at least one injection during the three months preceding the initiation of HCV therapy as reported by the patient. No additional screening was performed in order to assess recent drug use.

All patients with ongoing intravenous drug use were informed about harm-reduction measures and prevention of HCV transmission and reinfection upon each visit at the low-threshold facility (e.g. needle and syringe exchange programs).

Patients included in the DOT-group were followed up every six months to detect possible reinfections.

### Antiviral therapy

Patients received three fixed-dose combination tablets (each containing 100 mg of glecaprevir and 40 mg of pibrentasvir), administered orally once daily with food. Treatment duration was 8 weeks for patients without cirrhosis, 12 weeks for patients with compensated cirrhosis and 16 weeks for treatment-experienced GT3 patients, according to label. As recommended by current guidelines, patients with decompensated cirrhosis were not treated with G/P in order to avoid a potential toxicity of protease inhibitors in these patients[34].

If a patient missed ingestion of the drugs, the treatment period was prolonged for one day; i.e. our aim was that every patient received the originally planned number of tablets.

### Study endpoints

The primary endpoint of this study was SVR as defined by an HCV-RNA level <15 IU/ml 12 weeks after the end of treatment (SVR12). SVR12 rates were calculated for the intention-to-treat (ITT) population (including all patients who received at least one dose of antiviral medication) as well as for the modified ITT (mITT) population, defined as the ITT population excluding patients who did not achieve SVR12 for reasons other than virological failure; i.e. patients lost to FU and patients who died for reasons not related to therapy prior to SVR12. However, patients with poor compliance, who missed ingestion of their antiviral therapy on one or more days but were not lost to FU were included in the mITT analysis.

Secondary endpoints included adherence to antiviral therapy in the group of PWIDs treated according to the concept of DOT, the frequency of early termination of therapy and the occurrence rate of serious adverse events.

### Statistics

Patient characteristics are shown as median (IQR, interquartile range) for continuous variables or numbers (and percentage) of a certain characteristic for categorical variables. P-values were calculated using Student’s t-test, Wilcoxon-Mann-Whitney-test or Chi-Squared test, depending on the type of variable and the presence of normal distribution. In the primary efficacy analyses, two-sided 95% exact confidence intervals (CI) were calculated using the Clopper-Pearson method. The programs RStudio 1.0.44 for Windows and IBM SPSS Statistics, Version 25 for Mac were used for statistical analyses.

### Ethical considerations

The study protocol was approved by the institutional review board (Ethikkommission der Stadt Wien, EK 16-098-VK) and conducted according to the Declaration of Helsinki, Good Clinical Practice guidelines, and local regulatory requirements. Due to the strict anonymous analysis of patient data, the ethics committee waived the need for specific informed consent. However, to be absolutely sure to comply with data protection regulations, we obtained written informed consent from all patients.

## Results

### Study population

A total of 145 patients were included in this study; 62.8% of patients were male, the median age (IQR) was 41.1 (19.5) years, n = 4 (2.8%) were coinfected with HIV, n = 6 (4.1%) had cirrhosis, n = 135 (93.1%) were treatment-naïve, and GT1 in n = 82 (56.6%) followed by GT3 in n = 56 (38.6%) were the most frequently detected GT (Table 1).

**Table 1:** Baseline characteristics.

Variable	Overall	SS	DOT	p-value
n (%)	145 (100)	71 (49.0)	74 (51.0)	
OST [n (%)]	85 (58.6)	11 (15.5)	74 (100)	<0.0001
HIV [n (%)]	4 (2.8)	2 (2.8)	2 (2.7)	1
Sex [n (%)]				0.056
Male	91 (62.8)	39 (54.9)	52 (70.3)	
Female	54 (37.2)	32 (45.1)	22 (29.7)	
Age (years) [median (IQR)]	41.1 (19.5)	50.6 (20.3)	38.7 (12.5)	<0.0001
Fibrosis stage^a^ [n (%)]				0.792
F0/F1	90 (62.1)	44 (62.0)	46 (62.2)	
F2	34 (23.4)	15 (21.1)	19 (25.7)	
F3	15 (10.3)	8 (11.3)	7 (9.5)	
F4	6 (4.1)	4 (5.6)	2 (2.7)	
HCV genotype [n (%)]				0.313
1^b^	82 (56.6)	40 (56.3)	42 (56.8)	
- 1a	57 (39.3)	22 (31.0)	35 (47.3)	
- 1b	22 (15.2)	17 (23.9)	5 (6.8)	
2	1 (0.7)	0 (0.0)	1 (1.4)	
3	56 (38.6)	27 (38.0)	29 (39.2)	
4	5 (3.4)	4 (5.6)	1 (1.4)	
Not specified	1 (0.7)	0 (0.0)	1 (1.4)	
Treatment history				0.746
Naive [n (%)]	135 (93.1)	67 (94.4)	68 (91.9)	
Experienced [n (%)]	10 (6.9)	4 (5.6)	6 (8.1)	
Treatment duration (weeks) [median (IQR)]	8.0 (0.0)	8.0 (0.0)	8.0 (1.0)	0.328
8 weeks [n (%)]	115 (79.3)	59 (83.1)	56 (75.7)	
12 weeks [n (%)]	26 (17.9)	9 (12.7)	17 (23.0)	
16 weeks [n (%)]	4 (2.8)	3 (4.2)	1 (1.4)	

Abbreviations: SS, standard setting; DOT, directly observed therapy; OST, opioid substitution therapy; HIV, human immunodeficiency virus; IQR, interquartile range; HCV, hepatitis C virus.

^a^ according to transient elastography (TE; n = 135) or APRI (n = 10; applied ULN for AST: 35 IU/L (female) and 50 IU/L (male)):

F0/F1: TE 0–7.1 kPa or APRI ≤ 0.5

F2: TE 7.2–9.4 kPa

F3: TE 9.5–12.4 kPa

F4: TE ≥ 12.5 or APRI > 1.5

^b^ subtype not specified in n = 3 (2.1%)

**Fig 1:**
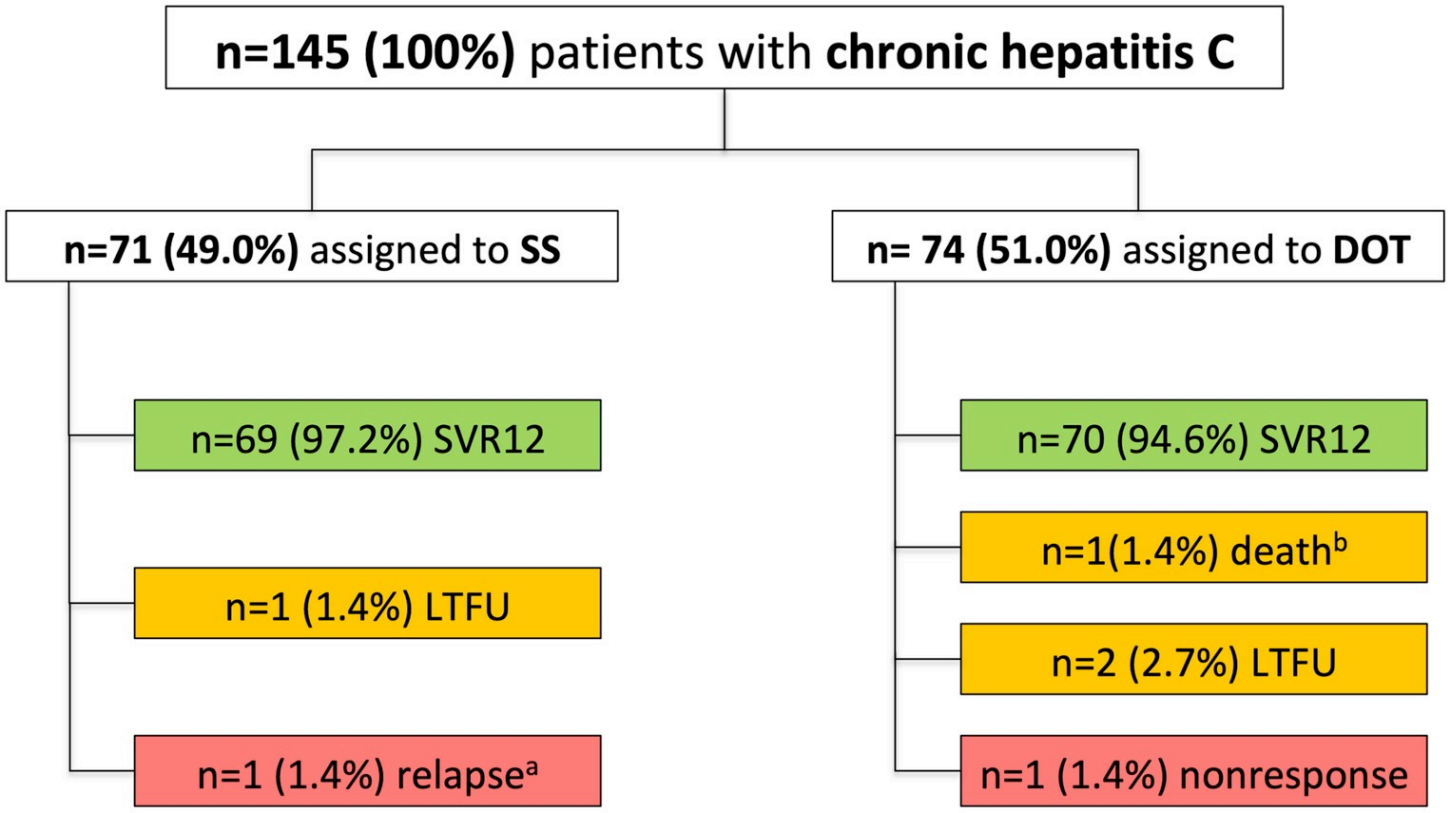
Flowchart of study patients. Abbreviations: SS, standard setting; DOT, directly observed therapy; SVR, sustained virologic response; LTFU, lost to follow-up. ^a^ at week 12 after end of therapy. ^b^ 16 weeks after end of treatment for reasons not related to therapy.

71 patients were treated in the standard setting (SS) of the outpatient clinic of a tertiary care center (Wilhelminen Hospital Vienna) and 74 PWIDs on OST received antiviral therapy with G/P at their pharmacy (n = 72) or at the Ambulatorium Suchthilfe Wien (n = 2)–always concomitantly with their OST according to the concept of DOT (Table 1, Fig 1). As compared to the SS-group, patients in the DOT-group were significantly younger and the percentage of men was slightly higher, as was the prevalence of GT1a. Most PWIDs included in the DOT-group were characterized by a very poor socioeconomic status: n = 64 (86.5%) were unemployed, n = 39 (52.7%) had no own housing, n = 49 (66.2%) had previously been imprisoned and only n = 29 (39.2%) reported to be living in a stable relationship; in addition, relevant psychiatric comorbidity was present in all of them; ongoing intravenous drug use was reported in n = 50 (67.6%) (Table 2). As compared to the DOT-group, the percentage of patients with employment, own housing and without a history of imprisonment was significantly higher in the SS-group.

**Table 2:** Socioeconomic characteristics of study patients at baseline.

Variable	Overall	SS	DOT	p-value
n (%)	145 (100)	71 (49.0)	74 (51.0)	
Employment status [n (%)]				0.007
Employed	32 (22.1)	22 (31.0)	10 (13.5)	
Unemployed	110 (75.9)	46 (64.8)	64 (86.5)	
Not known	3 (2.1)	3 (4.2)	0 (0.0)	
Own housing [n (%)]				<0.0001
Yes	90 (62.1)	55 (77.5)	35 (47.3)	
No	51 (35.2)	12 (16.9)	39 (52.7)	
Not known	4 (2.8)	4 (5.6)	0 (0.0)	
Living in stable relationship [n (%)]				0.069
Yes	65 (44.8)	36 (50.7)	29 (39.2)	
No	75 (51.7)	30 (42.3)	45 (60.8)	
Not known	5 (3.4)	5 (7.0)	0 (0.0)	
Criminal record				<0.0001
Imprisoned before	50 (34.5)	1 (1.4)	49 (66.2)	
Not imprisoned before	88 (60.7)	63 (88.7)	25 (33.8)	
Not known	7 (4.8)	7 (9.9)	0 (0.0)	

### Effectiveness of G/P therapy

**Fig 2:**
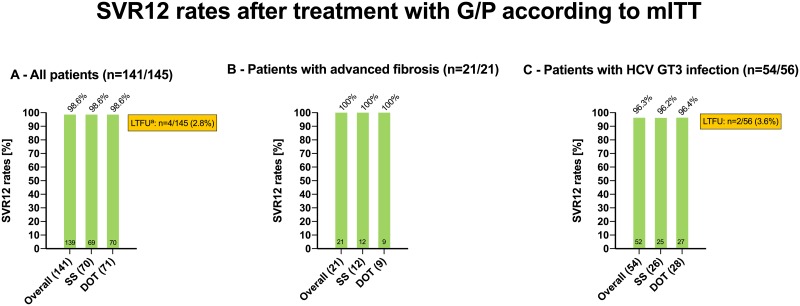
SVR12 rates after treatment with G/P according to mITT. Abbreviations: SVR, sustained virologic response; G/P, glecaprevir/pibrentasvir; mITT, modified intention-to-treat; SS, standard setting; DOT, directly observed therapy; LTFU, lost to follow-up; HCV, hepatitis C virus; GT, genotype. A—All patients (n = 141/145). ^a^ including one patient who died 16 weeks after end of therapy for reasons not related to treatment after showing a negative HCV-RNA PCR result three weeks after end of therapy. B—Patients with advanced fibrosis (n = 21/21). Advanced fibrosis was defined as F3-F4: ≥9.5 kPa according to transient elastography. C—Patients with HCV GT3 infection (n = 54/56).

In the SS-group SVR12 was achieved in 69 of 71 patients, resulting in an SVR12 rate of 97.2% (95% CI: 90.2–99.7%) according to ITT analysis. One patient was lost to follow-up (FU) after end of treatment response was documented, and one treatment-naïve patient with GT3 and F2 fibrosis experienced HCV relapse 12 weeks after end of antiviral therapy. After exclusion of the patient lost to FU, the SVR12 rate to G/P according to mITT analysis was 98.6% (95% CI: 92.3–100.0%) in the SS (Fig 2A–2C).

In the DOT-group SVR12 was achieved in 70 of 74 patients, resulting in an SVR12 rate of 94.6% (95% CI: 86.7–98.5%) according to ITT analysis. Two patients were lost to FU. One patient died 16 weeks after end of therapy for reasons not related to treatment after showing a negative HCV-RNA PCR result three weeks after end of therapy. However, the patient had missed the visit 12 weeks after cessation of therapy. One patient with GT3 infection and fibrosis stage F0/F1 who was treated for eight weeks did not respond to therapy: In fact the HCV-RNA levels of this patient did not significantly change during therapy. This patient showed up at the assigned pharmacy on a daily basis, yet we assume that the oral OST (as well as the antiviral medication) was not swallowed in order for the OST to be injected intravenously outside the pharmacy later. Unfortunately, due to very poor compliance to clinical visits HCV resistance testing could not be performed. All four patients without SVR12 reported ongoing IDU. After exclusion of the two patients lost to FU and the patient who died, SVR12 rate according to mITT analysis was 98.6% (95% CI: 92.4–100.0%) (Fig 2A–2C).

### Adherence to therapy and early termination of therapy

In the DOT-group 58 patients received their antiviral therapy together with OST on a once-daily basis, 14 on a once-weekly basis, one on a twice weekly and one on a thrice-weekly basis. Adherence to therapy was excellent: 70 of 74 patients (94.6%) did not miss a single scheduled date for ingestion of antiviral therapy together with OST; four patients missed 19, 7, 4 and 2 dates, respectively. Three of these four patients achieved SVR12; the patient who had missed 7 dates was lost to FU. Overall, only 32 out of 3,332 (0.96%) scheduled dates were missed by the patients. No patient discontinued treatment because of adverse events or lack of compliance.

### Serious adverse events

No serious adverse events related to antiviral therapy were observed in any of the 145 patients.

### Follow-up and HCV reinfections

So far, in the DOT-group, after a median FU (IQR) of 26.0 (34.0) weeks, one reinfection was recorded 71 weeks after end of therapy. The presumed mode of infection was needle sharing with a friend. Yet, 28 patients did not show up at the 24 weeks follow-up visit, therefore we cannot exclude that some reinfections were missed.

## Discussion

In Austria, treatment of hepatitis C is only performed at a limited number of authorized medical centers. After the stepwise reduction of national reimbursement restrictions for DAAs, a high percentage of HCV-infected patients have been successfully treated in Vienna[18]. Yet, HCV prevalence among PWIDs remains high. Especially PWIDs with ongoing IDU represent a relevant source of continuing HCV transmission and are thus an important target population for national HCV-elimination programs.

Various studies have shown that DAA therapy for HCV is safe in PWIDs, even in the presence of ongoing IDU and/or OST, and leads to excellent cure rates that do not deviate much from patients without a history of IDU [35,36]. We hypothesized that directly observed DAA therapy along with OST under the supervision of medical staff at local pharmacies or low-threshold facilities would further increase DAA adherence and improve HCV treatment outcomes in PWIDs on stable OST with or without ongoing IDU. The Ambulatorium Suchthilfe Wien, a low-threshold drug treatment facility in Vienna, offers integrative management combining HCV screening and therapy for PWIDs by providing hepatological, psychiatric and social support services as well as OST distribution in a multidisciplinary setting. Indeed, we have previously demonstrated that the concept of DAA-DOT alongside OST is feasible in patients at high risk for non-adherence to therapy[32,37]. Based on these considerations and the available data, PWIDs on stable OST were included in this study and received directly observed HCV therapy with G/P. The included PWIDs showed excellent adherence to therapy with only a very limited number of scheduled DAA-ingestions being missed and, most importantly, an SVR rate of 98.6%—which was in fact similar to the SVR rate of 98.6% achieved in patients with presumed “excellent compliance” with G/P self-administration. The observed excellent safety profile of G/P as well as the high SVR rates that did not relevantly deviate from the treatment outcomes in patients without a history of IDU go in line with literature and support the recommendations of broad access to HCV treatment [35,36].

From our results we conclude that directly observed DAA therapy along with OST might be an efficient strategy to achieve SVR rates comparable to patients without a history of IDU in PWIDs with high risk of non-adherence to DAA therapy even in the presence of ongoing IDU. This concept represents a relevant public health strategy in the attempt to eliminate hepatitis C in Austria.

In accordance with previous studies, SVR rates after treatment with G/P were excellent and no serious adverse events related to therapy occurred[20,22–25,38]. During follow-up, only one HCV reinfection was detected which might be due to the integrated management of PWIDs at our low-threshold facilities in Vienna that offer counseling and syringe exchange services along with medical treatment for PWIDs[39].

We present results of a monocenter study including part of the Austrian PWIDs population: n = 74 PWIDs with suspected poor compliance out of about 6.500 PWIDs who live in Vienna and are included in the nationwide OST program were treated according to the DOT-concept. The outcome of these patients was compared to a cohort of patients with presumed excellent compliance (including 11 patients on OST) who were treated according to the SS of HCV care. The specific characterization of the real-world treatment outcome of G/P in difficult-to-treat PWIDs with and without ongoing IDU using the setting of DAA-DOT alongside OST represents the major strength of this study. Yet, the assignment of PWIDs to either DAA self-administration or to DOT with G/P at an OST distribution facility was based on subjective assessment of presumed compliance by the study physicians and therefore poses a potential limitation. Another limitation is that some patients did not attend their OST distribution facility on a daily basis but only once, twice or thrice a week and received their corresponding OST and DAA doses for interim self-administration, according to the subjective estimation of the study staff assessing the patients’ individual risk for non-adherence to therapy. Overall, this study focuses on PWIDs with an unfavorable prognosis assumed by ongoing IDU, previous imprisonment and unstable socioeconomic factors. The inclusion in an OST program was considered a positive prognostic factor and was used as a connecting point for HCV treatment. Aside from this PWIDs population that is already linked to care, a relevant number of PWIDs in Vienna perform IDU and are not included in an OST program, most of whom would not show compliant behavior concerning medical treatment. HCV infected patients belonging to this subgroup of "chaotic PWIDs" currently cannot be treated because a regular ingestion of drugs cannot be achieved.

In order to address these especially difficult to treat patients, we are planning to introduce a peer-based HCV screening and treatment program for PWIDs in Vienna. The project shall focus on PWIDs who have successfully been cured from HCV or are currently on treatment bringing their friends along to the diagnostic facility and thereby linking HCV infected PWIDs to treatment who would otherwise not attend a medical facility.

To enhance the relevance of our results, further studies including multicenter studies investigating a broader variety of patients are needed. Yet, our results add important and encouraging information to the still scarcely available real-life data on HCV therapy in PWIDs with ongoing IDU.

In conclusion, our results underline the safety and the excellent efficacy of G/P in a large real world cohort of PWIDs with mostly ongoing IDU. The concept of tailored HCV therapy using DOT with DAA alongside OST may represent a key measure in order to contribute to the HCV elimination target in difficult-to-treat PWIDs on OST with a high risk of non-adherence to DAA therapy.

## Supporting information

S1 Dataset (XLSX)

## Abbreviations

CI: confidence interval
DAA: directly acting antivirals
DOT: directly observed therapy
FU: follow-up
G/P: glecaprevir/pibrentasvir
GT: genotype
HBV: hepatitis B virus
HCC: hepatocellular carcinoma
HCV: hepatitis C virus
HIV: human immunodeficiency virus
IDU: injection drug use
IQR: interquartile range
ITT: intention to treat
LS: liver stiffness
mITT: modified intention to treat
OST: opioid substitution therapy
PWIDs: people who inject drugs
RNA: ribonucleic acid
SS: standard setting
SVR: sustained virologic response
WHO: World Health Organization
